# Effect of vitamin and mineral supplementation on nutritional status in children with chronic kidney disease: Protocol for a systematic review and meta-analysis

**DOI:** 10.1097/MD.0000000000031518

**Published:** 2022-10-28

**Authors:** Archi Mutsuddi, Jyoti Das, Symom Tashrik, Rifat Ara, Mohammad Delwer Hossain Hawlader

**Affiliations:** a Department of Public Health, North South University, Dhaka, Bangladesh; b Public Health Professional Development Society (PPDS), Dhaka, Bangladesh; c Infectious Disease Division, icddr,b, Dhaka, Bangladesh

**Keywords:** children, chronic kidney disease, minerals, vitamins

## Abstract

Persistent kidney illness in children and those on dialysis therapy, risk of vitamin and mineral deficiencies due to abnormal renal metabolism, anorexia, inadequate intake, poor gastrointestinal absorption, drug-nutrient interaction, and dialysis-related losses. Adequate nutritional management is essential to achieve and maintain an optimal nutritional status for the usual pattern of growth, sexual and neurocognitive development, metabolic abnormalities, and ultimately reducing the risk of chronic mortality and morbidity in adulthood. The purpose of this paper is to describe a protocol for a systematic review to assess the effects of vitamin and mineral supplementation in children with chronic renal disease.

**Methods:**

This systematic review protocol is developed according to the Preferred Reporting Item for Systematic Review and Meta-Analysis Protocols (PRISMA-P) as well as the Cochrane group guidelines. Comprehensive searching for all possible relevant works of literature- such as PubMed, Google Scholar, MEDLINE, Embase, Web of Science, Cochrane Central Register of Controlled Trials (CENTRAL), Science-Direct, Scopus, Research-Gate, Clinical Trials for all randomized controlled studies, full paper articles, and articles written in English will be considered. The primary outcome of this review will be measuring any changes (such as changes in body mass, BMI, and overall Z-score) in the nutritional status of the children (age < 18 years) with chronic kidney disease following vitamin and mineral supplementations. This review will help better understand the effects of vitamin and mineral supplementation to improve nutritional status in CKD children and will create a guideline to determine the applicability of these interventions in different feasible settings.

**Conclusion:**

The systematic review protocol has been evaluated and approved by the institutional review board of North South University. Finding will be shared using traditional approaches, including scientific presentations, open-access peer-reviewed platforms.

**PROSPERO registered number:**

CRD42022341339

## 1. Introduction

Chronic kidney disease (CKD) in children has become increasingly recognized as a global public health concern and a significant contributor to morbidity and mortality over the last decade.^[1]^ While the burden of CKD is well recognized in industrialized nations, growing data suggests that the burden of CKD may be significantly more significant in underdeveloped countries.^[1]^ According to the NAPRTS (North American Pediatric Renal Transplant Cooperative Society), congenital anomalies of the kidney and urinary tract and hereditary nephropathies account for approximately two-thirds of all cases of CKD in children in developed countries. In contrast, acquired causes are more common in developing countries.^[2]^

Although there are parallels between the CKD populations of adults and children, children show specific characteristics that are not present in adults, such as diseases affecting their growth.^[1]^ Children with CKD frequently experience significant development retardation, which affects up to 35% of this population before developing the end-stage renal disease.^[1]^ In addition to that adequate nutrition and routine evaluations are necessary to meet the children’s vitamin and mineral needs and stop the onset of protein-energy malnutrition.^[1]^ CKD patients are at risk of micronutrient deficiencies. It may be due to the overall decrease in nutritional intake, restrictions in diet, poor intestinal absorption, inflammatory state, metabolic acidosis, and dialysate losses. Ultimately resulting in comorbidities such as anemia, cardiovascular disease, and metabolic imbalances.^[1]^

Vitamin D insufficiency is commonly found in children with chronic kidney disease (CKD), and it relates to mineral and bone problems.^[3–4]^ Furthermore, there is evidence that vitamin D may play a role in various other areas, including cardiovascular disease, immunological function, and the prevention of autoimmune illnesses and some cancers.^[5–6]^ A range of problems in mineral metabolism leads to bone damage in patients with chronic kidney disease (CKD).^[7,8]^ Nutritional vitamin D deficiency is common in patients with CKD, with a prevalence of 60% to 80% in pre-dialysis children.^[3,9–11]^ Observational studies reveal that vitamin D therapy may provide considerable advantages for dialysis patients.^[12,13]^ In patients with CKD stages 2 to 5, guidelines advise measuring blood 25-hydroxy vitamin D every 2 years or annually and treating dietary vitamin D deficiency. These recommendations preserve vitamin D deficiency below 30 ng/mL.^[14,15]^

Hepcidin, an iron-regulatory protein, has emerged as a potentially modifiable mediator of chronic kidney disease (CKD) anemia, including the erythropoiesis-stimulating agent-resistant anemia.^[16,17]^ Through the posttranslational inhibition of cell-membrane ferroportin expression, hepcidin controls intestinal iron absorption and body iron distribution.^[18]^ When ferroportin is bound by hepcidin, it is internalized and destroyed, which reduces intestinal enterocytes’ ability to absorb dietary iron and prevents the release of intracellular (in hepatocytes and monocytes) iron stored in ferritin for use in erythropoiesis.^[19]^ Adults and children with CKD and those on dialysis have higher hepcidin levels.^[20]^A transcriptional novel regulator of hepcidin synthesis is vitamin D.^[21]^

Chronic renal failure (CRF) is frequently accompanied by accelerated vascular disease which is a significant cause of mortality and morbidity. Elevating circulating homocysteine levels in CRF is one possible mechanism for endothelial damage. Homocysteine is a distinct risk factor for developing CRF, and it is more pervasive in dialysis patients than in people with predefined cardiovascular risk factors.^[22]^ In numerous populations, folic acid has been demonstrated to reduce homocysteine levels. Children were explicitly recruited to minimize the impact of confounding factors and so represent a clinical model of uremic influences on the artery wall, as well as to determine whether early identification might have more significant vascular benefits than adults.^[23]^

Zinc is one of the most significant and necessary trace elements needed by all living for a variety of physiologic processes, with three vital biological roles: catalytic, structural, and regulatory.^[24,25]^ Zinc, an essential cofactor, influences the expression and activity of many enzymes, transcription factors, and regulatory proteins. At least 3000 proteins and cellular components depending on their structure and function. It is essential for the immune system, cellular growth, proliferation, apoptosis, and many zinc-binding proteins’ activity.^[24]^ Zinc is known to control growth, immunity, and neurological development. If it is deficient, it can impair the growth of various organs, including the brain, lungs, skeleton, kidneys, and heart.^[26]^ Zinc deficiency is associated with CKD and exacerbates renal problems.^[27]^ The National Kidney Foundation KDOQI recommends that both children and adults should get the dietary reference intakes (DRI) for zinc even though the needs for zinc in CKD patients have not been determined. KDOQI also advises that supplementation to be given if necessary, and that intake for the newborn is 4.0 to 5.0 mg/d and for children, 5.0 to 9.5 mg, needs to monitor every 4 to 6 months,^[14]^ but these doses may not be enough for CKD children in developing countries.

The phosphate ester derivative pyridoxal 5′-phosphate, which is the biologically active form of vitamin B6, is required for iron incorporation into proto porphyrin, the last step in the synthesis of heme. Vitamin B6 deficiencies are usually associated with inflammation, as demonstrated in conditions like chronic kidney disease (CKD), particularly those requiring dialysis.^[28]^ A clinical sign of advanced kidney failure is erythropoietin insufficiency, especially in children receiving continuous dialysis. The erythropoietin-stimulating agent increases the rate of erythropoiesis and the need for iron, increasing the intake of vitamin B6.^[29]^ Vitamin B6 is an essential cofactor for erythropoiesis, and it has been reported to be deficient in hemodialysis patients.^[30]^ While uncommon in the general community, vitamin B6 insufficiency has been found to occur 24% to 56% frequently among juvenile hemodialysis patients. Therefore, initiating vitamin B6 supplementation in these individuals may be beneficial.^[31]^ There are no established guidelines for monitoring vitamin B6 levels or choosing the appropriate vitamin B6 supplement for treating vitamin B6 deficiency in pediatric hemodialysis patients.^[32]^ The scientific community is more interested than ever in examining the role and influence of vitamin B12 in CKD since impaired vitamin B12 metabolism is considered a nontraditional risk factor for poor outcomes associated with pediatric CKD.^[33]^

In chronic kidney disease (CKD), iron deficiency anemia is a severe problem that raises mortality among children. There are two types of iron deficiency (ID): absolute ID, which is characterized by a decrease in body iron reserves, and functional ID, which is characterized by normal or even higher total body iron levels but insufficient iron supply to the bone marrow.^[34]^ Iron deficiency anemia affects mortality in children with CKD.^[35]^ The purpose of this study is to look at the influence of vitamin and mineral supplements on nutritional aspects in children with chronic renal disease. This study aims to review the influence of vitamin and mineral supplements on nutritional aspects in children with chronic renal disease.

## 2. Methods and materials

### 2.1. Protocol

The systematic review will be conducted according to the norms of the Preferred Reporting Item for Systematic Review and Meta-Analysis Protocols (PRISMA-P) as well as the Cochrane group guidelines. PRISMA-P checklist has been attached. The PROSPERO registered number is CRD42022341339. The PRISMA-P checklist for systematic review protocol has been attached as a supplementary file.

### 2.2. Eligibility criteria

The studies will be considered based on the criteria outlined below.

### 2.3. Participants

This review will include studies targeting only children (age < 18 years) with chronic kidney disease

#### 2.3.1. Comparator.

A comparison will be made between the group with the intervention and either the control group or no intervention group or placebo group.

### 2.4. Intervention

This systematic review will consider studies which are assessing the effects of nutritional intervention on the nutritional status of children (below 18 years) with chronic kidney diseases. The intervention will include vitamin and mineral supplements such as Vitamin B1, B2, B6, B12, Vitamin D, Vitamin C, folic acid, etc. and for minerals Iron, Zinc etc.

### 2.5. Outcome

#### 2.5.1. Primary outcome.

The primary outcome of this review will be measuring any change in nutritional status of the children with chronic kidney disease following vitamin and mineral supplementations. The measurement will be taken using body mass, BMI and overall Z-score.

#### 2.5.2. Secondary outcome.

Secondary outcome will be assessing the effectiveness of the interventions, measuring any change in Markers of bone and mineral metabolism, effect on bone lesion, reduce plasma homocysteine level, delays onset of Secondary hyperparathyroidism, effect on serum Hepcidin level.

#### 2.5.3. Study setting.

Studies that were conducted in hospital settings and where participants were recruited from both the outpatient pediatric nephrology unit; CKD transplant, peritoneal and hemodialysis clinics of the respective hospitals will be included.

### 2.6. Inclusion criteria

1. Randomized control trials
2. All the full paper articles
3. Published in the English language

### 2.7. Exclusion criteria

1. All other non-randomized control trials, pre-post studies, case-control studies, descriptive studies, cohort studies, cross-sectional studies, and case reports will be excluded.
2. Any abstract, or conference paper.

#### 2.7.1. Information sources.

A comprehensive search strategy will be developed based on the research question to search all the relevant literature. It will be conducted by independently by the reviewers from the following databases- PubMed, Google Scholar, MEDLINE, Embase, Web of Science, The Cochrane Library (Cochrane Central Register of Controlled Trials (CENTRAL), Science-Direct, Scopus, Research-Gate, Clinical Trials, etc. Keywords and Search terms will be adapted for use in different bibliographic databases in combination with database-specific filters.

#### 2.7.2. Search strategy.

A comprehensive search strategy will be developed through a systematic approach using the keywords and “MeSH” terms according to PICO (population, intervention, comparison, outcome), for PubMed. This will be adapted for other bibliographical databases in combination with database-specific filters. Wild cards and truncations will be used in search terms where necessary. Studies up to date will be searched for and before the final analysis, all the search strategies will be rerun to seek new articles to include. According to PICO, the essential terms implemented in this review are listed in Table 1. A comprehensive search strategy in PubMed format is listed in Table 2.

**Table 1:** PICO table for the preparation of search strategy.

Population	AND Intervention	AND Comparison	AND Outcome	Types of studies (filter)
-Children OR -Infant OR -Toddler OR -Kids OR -Adolescents OR -Teenagers OR -Pediatrics	-Vitamins OR -“Vitamin B1” OR -“Vitamin B2” OR -“Vitamin B6” OR -“Vitamin B12” OR -“Vitamin D” OR -“Vitamin C” OR -“Folic acid” OR -Minerals OR -Iron OR -Zinc	-Efficacy OR -Effectiveness OR -Efficiency OR -Potency OR -Usefulness	-“Chronic kidney disease” OR -CKD OR -“Chronic renal disease” OR -“Chronic renal failure”	RCTs “Randomized controlled trials”

**Table 2:** Comprehensive search strategy: PubMed format.

1	“child”[MeSH Terms] OR “child”[All Fields] OR “children”[All Fields] OR “child s”[All Fields] OR “children s”[All Fields] OR “childrens”[All Fields] OR “childs”[All Fields] OR (“Infant”[MeSH Terms] OR “Infant”[All Fields]) OR “toddler”[All Fields] OR “Kid”[All Fields] OR ((“adolescent”[MeSH Terms] AND “adolescent”[All Fields]) OR “teen”[All Fields]) OR (“pediatrics”[MeSH Terms] OR “pediatrics”[All Fields] OR “pediatric”[All Fields] OR (“pediatrics”[All Fields] OR “pediatric”[All Fields]))
2	“Vitamins”[All Fields] OR “Vitamin B1”[All Fields] OR “Vitamin B2”[All Fields] OR “Vitamin B6”[All Fields] OR “Vitamin B12”[All Fields] OR “Vitamin D”[All Fields] OR “Vitamin C”[All Fields] OR “Folic acid”[All Fields] OR “Minerals”[All Fields] OR “Iron”[All Fields] OR “Zinc”[All Fields]
3	“effect”[All Fields] OR “effects”[All Fields] OR “effecting”[All Fields] OR “effective”[All Fields] OR “effectively”[All Fields] OR “effectiveness”[All Fields] OR “effectivenesses”[All Fields] OR “effectives”[All Fields] OR “effectivity”[All Fields] OR “effectivities”[All Fields] OR “efficacy”[All Fields] OR “efficacies”[All Fields] OR “efficacious”[All Fields] OR “efficaciously”[All Fields] OR “efficaciousness”[All Fields] OR “efficiency”[MeSH Terms] OR “efficiency”[All Fields] OR “efficiences”[All Fields] OR “efficiencies”[All Fields] OR “efficient”[All Fields] OR “efficiently”[All Fields] OR “efficients”[All Fields] OR “potency”[All Fields] OR “potencies”[All Fields] OR “useful”[All Fields] OR “usefulness”[All Fields]
4	“Chronic kidney disease”[All Fields] OR “CKD”[All Fields] OR “Chronic renal disease”[All Fields] OR “Chronic renal failure”[All Fields]
5	1 AND 2 AND 3 AND 4

#### 2.7.3. Data extraction.

According to inclusion and exclusion criteria, two independent reviewers will first screen the title and abstract. After that, retrieval of eligible studies will be done for final review and Mendeley software will be used for screening. At this stage, if any confusion or disagreement arises regarding inclusion between the reviewers, then it will be resolved by the third reviewer. Repeated articles and multiple publications from the same study will be excluded and all the reasons for exclusion will be reported. PRISMA flow diagram will describe how the studies were found and included for review by applying the customized inclusion and exclusion criteria. A standardized form will be created using Microsoft Excel to extract data from the included studies. It will be used to assess the study quality and for evidence synthesis. The extracted information will include: study settings; study population; participant’s demographics and baseline characteristics; details of the interventions and control conditions; study methodology; recruitment process and study completion rates; outcomes and times of measurement; indicators of acceptability to users; information for the assessment of the risk of bias, etc.

#### 2.7.4. Data synthesis.

After the full-text screening of the articles, an exploration of the available interventions will be conducted and mapped accordingly. A narrative synthesis focusing on the targeted population (both control/placebo and intervention group), intervention description, and the outcome will be done from the included studies as it helps to compare the similarities and dissimilarities of the selected homogenous studies and helps to arrange them together. For continuous outcomes, we will record the mean change from baseline and SD with 95% CI and consider using the standardized mean difference (with 95% CI) as in some cases same outcomes are measured on different scales. For dichotomous data, the measure of the effect size will be presented as OR an RR with the 95% Confidence Interval (CI). If similar interventions for the treatment group, similar participant groups, and similar outcome measures could be derived from the included studies, then pooled treatment effects will be estimated through meta-analyses. The heterogeneity of the studies in effect measures will be assessed by both the Chi-square test and the *I*^²^ statistics. A *P* value < .1 and *I*-squared over 50% indicate a high level of heterogeneity. A fixed-effect (or random effects) model will be employed when heterogeneity is absent (or present). It is anticipated that there will be limited scope for meta-analysis secondary to the anticipated small number of existing trials and range of outcome measures reported. If meta-analysis is not appropriate, heterogeneity will be evaluated by describing and comparing the study samples, methods, and designs across studies. Sensitivity analysis will also be conducted based on the study quality. In addition, a funnel plot will be generated through the review manager software (RevMan) for assessing the potential publication bias for each study.

#### 2.7.5. Quality assessment.

The risk of bias in the included studies will be assessed independently by two reviewers. It will be done based on seven evidence-based domains: random sequence generation, allocation concealment, blinding of participants and personnel, blinding of outcome assessment, incomplete outcome data, selective reporting, and other biases. They will be categorized into the low risk of bias, high risk of bias, and unclear depending on the review author’s judgments. Disagreements between the reviewers will be discussed and resolved by consensus. However, insights from the third reviewer will be sought if necessary.

#### 2.7.6. Analysis of subgroups or subsets.

If the necessary data are available, Subgroup analyses may include: Child characteristics including age (under 5 years vs over 5 years) and gender (male vs female), Nutritional status at inclusion (mild, moderate, and severe undernutrition), Type of intervention (vitamins & minerals), Type of kidney disease (glomerular, non-glomerular) and will be included in meta-regression models to evaluate the effect of participant characteristics on the effectiveness of the interventions.

### 2.8. Ethics and dissemination

Ethical approval will not be required for this review as the data used will be extracted from already published studies with publicly accessible data. We will share our findings using traditional approaches, including scientific presentations, open-access peer-reviewed platforms, and appropriate government and public health agencies.

## 3. Discussion

Childhood and adolescence are the most vital periods for a child’s growth and development, and the provision of adequate nutrients is essential. Children with CKD and those on dialysis therapy risk vitamin and mineral deficiencies due to abnormal renal metabolism, anorexia, inadequate intake, poor gastrointestinal absorption, drug-nutrient interaction, and dialysis-related losses.^[36–39]^ Moreover, regular evaluation of nutritional status and adequate nutrition are critical components in managing children with all stages of CKD. The primary focus of nutritional management for children with renal insufficiency is to prevent the development of Protein-energy Malnutrition and meet the patient’s vitamin and mineral needs.^[40]^ According to the 2008 update of the KDOQI Clinical Practice Guideline for Nutrition in Children with CKD, nutritional care is essential to achieve and maintain an optimal nutritional status for the usual pattern of growth; sexual and neurocognitive development, avoidance of chronic uremic toxicity, metabolic abnormalities, and malnutrition and reduce the risk of chronic mortality and morbidity in adulthood.^[40–42]^

Short stature and significant growth retardation are well-recognized complications of childhood CKD. Furthermore, short stature is associated with increased morbidity and mortality. Wong et al reported a 14% increase in death risk for each 1 SD score decrease in height among children with end-stage renal disease. Children with height SD scores less than −2.5 at dialysis initiation were found to have 2.07 (95% CI: 1.53, 2.77) times the risk of death and to spend 0.22 more days per month in the hospital compared with those with height SD scores more than −2.5.^[42]^ Body mass index (BMI), an indicator of muscle mass, is also related to the adverse outcome: there is a U-shape association between BMI and death. Increased risk of infection in malnourished patients could be part of the explanation for their increased mortality. Inflammation and acidosis exert competitive effects on adequate nutrition and expected growth, which may be important outcome risk factors.^[41]^

Children with CKD and those on dialysis therapy risk vitamin and mineral levels or functional alterations. B complex vitamins are grouped, and one of the essential functions of water-soluble vitamins such as vitamins B6, B12, and folic acid is to work together with iron to prevent anemia. Additional B vitamins such as thiamin, riboflavin, pantothenic acid, and niacin help change the consumed foods into energy. Vitamin C helps wounds and bruises heal faster and may help prevent infections. Calcium, along with vitamin D, helps to keep bones healthy. Moreover, chronic uremia causes low levels of plasma calcium and high phosphate levels that increase the synthesis and secretion of parathyroid hormone, causing secondary hyperparathyroidism.^[43]^

Most data are derived from studies in adult populations and a limited number of studies conducted on children on nutrition in all stages of CKD. Moreover, evidence-based quality studies related to this issue are less due to the small sample size, few randomized controlled trials, and the lack of information for both short- and long-term clinical outcomes.^[40]^

We hope that the systematic review will bring results that allow us to understand the effects of vitamin and mineral supplementation to improve nutritional status in children with chronic kidney disease and will create a guideline to determine the applicability of these interventions in different feasible settings. This review will also provide the opportunity for further research by revealing the research gap on this issue. We foresee several potential limitations with this systematic review, such as heterogeneity of clinical outcomes and this protocol considering only English language articles. We might miss the evidence published in Chinese, Latin, and other languages if any.

## Author contributions

**Study conceptualization:** All the authors contributed to the conceptualization.

**First draft of the protocol:** Archi Mutsuddi, Jyoti Das, Symom Tashrik, Rifat Ara.

**Protocol review and finalization:** Rifat Ara, Mohammad Delwer Hossain Hawlade.

**Literature search and screening:** Archi Mutsuddi, Rifat Ara, Symom Tashrik.

**Data abstraction:** Archi Mutsuddi, Rifat Ara, Jyoti Das.

**Quality assessment:** Archi Mutsuddi, Rifat Ara.

**Data synthesis and analysis:** Archi Mutsuddi, Rifat Ara, Jyoti Das.

**First draft of the article:** Archi Mutsuddi, Rifat Ara, Jyoti Das.

**Review and finalization:** Mohammad Delwer Hossain Hawlade.

**Final draft of the article:** All the authors will review and approve the final script.

The senior author is the guarantor of this review.
